# Revisits, readmissions, and outcomes for pediatric traumatic brain injury in California, 2005-2014

**DOI:** 10.1371/journal.pone.0227981

**Published:** 2020-01-24

**Authors:** Renee Y. Hsia, Rebekah C. Mannix, Joanna Guo, Aaron E. Kornblith, Feng Lin, Peter E. Sokolove, Geoffrey T. Manley

**Affiliations:** 1 Department of Emergency Medicine, University of California, San Francisco, California, United States of America; 2 Philip R. Lee Institute for Health Policy Studies, University of California, San Francisco, California, United States of America; 3 Division of Emergency Medicine, Boston Children’s Hospital, Boston, Massachusetts, United States of America; 4 Department of Emergency Medicine, Brigham and Women’s Hospital, Boston, Massachusetts, United States of America; 5 Department of Biostatistics and Epidemiology, University of California, San Francisco, California, United States of America; 6 Brain and Spinal Injury Center (BASIC), University of California, San Francisco, California, United States of America; 7 Department of Neurological Surgery, University of California, San Francisco, California, United States of America; Monash University, AUSTRALIA

## Abstract

Long-term outcomes related to emergency department revisit, hospital readmission, and all-cause mortality, have not been well characterized across the spectrum of pediatric traumatic brain injury (TBI). We evaluated emergency department visit outcomes up to 1 year after pediatric TBI, in comparison to a referent group of trauma patients without TBI. We performed a longitudinal, retrospective study of all pediatric trauma patients who presented to emergency departments and hospitals in California from 2005 to 2014. We compared emergency department visits, dispositions, revisits, readmissions, and mortality in pediatric trauma patients with a TBI diagnosis to those without TBI (Other Trauma patients). We identified 208,222 pediatric patients with an index diagnosis of TBI and 1,314,064 patients with an index diagnosis of Other Trauma. Population growth adjusted TBI visits increased by 5.6% while those for Other Trauma decreased by 40.7%. The majority of patients were discharged from the emergency department on their first visit (93.2% for traumatic brain injury vs. 96.5% for Other Trauma). A greater proportion of TBI patients revisited the emergency department (33.4% vs. 3.0%) or were readmitted to the hospital (0.9% vs. 0.04%) at least once within a year of discharge. The health burden within a year after a pediatric TBI visit is considerable and is greater than that of non-TBI trauma. These data suggest that outpatient strategies to monitor for short-term and longer-term sequelae after pediatric TBI are needed to improve patient outcomes, lessen the burden on families, and more appropriately allocate resources in the healthcare system.

## Introduction

Children ages 0 to 14 years account for over half a million emergency department (ED) visits for traumatic brain injury (TBI) annually in the United States (US).[1] TBI remains the leading cause of death and permanent disability in children, yet the majority of TBI cases (75% to 90%) are concussions or other forms of mild TBI.[2,3] The most recent (2017) report by the US Centers for Disease Control and Prevention (CDC) found an increase in pediatric TBI-related ED visits but a decrease in hospitalizations and deaths from 2007 to 2013,[1] which could suggest an increase in the incidence of mild TBI or a greater willingness to seek emergency care for less severe pediatric head injuries.

Current evidence on longer-term outcomes suggests that children with TBI may also have longer-term healthcare needs, [4–6] and children may be more vulnerable to longer-term negative outcomes in a manner different from adults sustaining similar injuries.[7–9] Other studies have also found significant unmet needs for children after TBI,[10–13] many of which are for cognitive and behavioral services. Furthermore, the actual burden of pediatric TBI on the healthcare system is most likely underestimated since studying outcomes after pediatric TBI is challenging; symptoms may be subtle or delayed, with sequelae that range from neuropsychiatric disorders[14] to gastrointestinal complaints,[15] which may potentially underestimate ED diagnoses and miss longer-term effects of TBI.[16] Identifying the unmet healthcare needs of pediatric patients with TBI, from mild to severe, may not only improve longer-term health outcomes but also lessen the burden on family members and caretakers.[17]

The lack of any standardized system of care for adult or pediatric patients with TBI in the United States [18] may contribute to increased emergency care utilization as EDs serve as a safety-net for many types of unmet healthcare needs. Most of the traditional literature on pediatric TBI focuses on hospitalized patients, rather than a more comprehensive view of outpatient visits to the ED that may result in direct discharge. In addition, longer-term healthcare utilization, more specifically, ED revisits and readmissions have not been widely studied for pediatric TBI. To fill this current gap in the literature, we used a longitudinal approach to retrospectively evaluate ED revisits, readmissions, and 1-year mortality for pediatric patients with TBI of all types of severity who were discharged from California hospitals from 2005 to 2014. Additionally, we compare our group of pediatric TBI patients to a referent group of pediatric trauma patients without TBI as other studies have done.[6,19,20] Our study uniquely assesses ED revisits and readmission outcomes using a longitudinal, large administrative database, which may better capture the need for follow-up care or lack of access to follow-up care after TBI, and can inform the organization of systems of care for improving longer-term outcomes in children with TBIs of all types of severity.

## Methods

(See S1 Methods for fuller description).

### Data

We used non-public patient-level data from the California Office of Statewide Health Planning and Development (OSHPD). This database contains information reported by all non-federal, general acute-care hospitals in California, including non-anonymized information such as date of admission, patient demographics, co-morbidities, diagnostic and procedural information, Injury Severity Scores (ISS), external cause of injury codes,[21] disposition, and total charges for inpatient admissions. We grouped patients into categories of mild (ISS< 9), moderate (ISS 9–16), or severe (ISS> 16)[22,23] (Department of Public Health and Health Services, Montana). Using vital statistics data we tracked mortality until 2011 (the last year for which linked death files were available). We linked these data with the OSHPD utilization and financial files, which contain hospital-level information such as trauma center status. To calculate visit rates and demographic data, we obtained population data using the US Census, American Community Survey, California Department of Finance, and Current Population Survey.

### Selection of participants

Our population included all pediatric patients aged ≤17 years who were seen at California EDs and hospitals and received a trauma diagnosis. To identify the target TBI population, we selected patients with any diagnosis using the International Statistical Classification of Disease, Ninth Revision (ICD-9) diagnosis codes from the 2017 CDC TBI report: 800.xx, 801.xx, 803.xx, 804.xx, 850.xx– 853.xx, 854.0, 854.1, 950.1–950.3, 959.01, and 995.55.[1] We identified trauma patients with codes 805.xx– 959.xx, exclusive of those coded as late effects of injury (905.xx– 909.xx), superficial injuries (910.xx– 919.xx), or foreign bodies (930.xx– 939.xx). Patient visits were classified as TBI (visits with any TBI diagnosis) or Other Trauma (trauma visits without any TBI diagnosis).

### Linkage of encounters

We linked all visits with the same record linkage number to track the same patient over time. The first visit for each patient was considered the index visit, and if multiple visits occurred within 1 day of each other, we did not consider these as separate events given that these could represent ED-to-ED or ED-to-inpatient transfers. TBI and Other Trauma index visits were mutually exclusive, i.e., if a first TBI visit was a revisit of an Other Trauma visit, it was not counted as an index visit. Similar to prior studies identifying ED revisits,[24–26] we categorized all subsequent visits as revisits and identified readmissions as revisits that resulted in hospitalization.

### Outcomes measured

The primary outcomes included revisits (all ED visits, not only those resulting in admission) and readmissions. We chose to evaluate subsequent revisits and readmissions for any diagnosis after the initial index TBI (or non-TBI trauma), because post-TBI symptoms in children can present atypically, and thus TBI-related healthcare use is not always easy to identify.

Secondary outcomes included: disposition; mean and median inpatient charges for the first year following the index TBI; and mortality (ED mortality, in-hospital mortality, and out-of-hospital 1-year mortality).

### Statistical analyses

We used descriptive statistics to compare characteristics of TBI patients to Other Trauma patients as a whole sample and as visit rates by year (calculated per 100,000 residents/year, where residents refer to the population in the specified demographic group for California). We tracked changes in disposition from the ED and inpatient setting for TBI and Other Trauma patients separately. To assess secular patterns, we estimated negative binomial models for the numbers of visits, with robust standard errors to account for clustering by hospital, calendar year of admission as a categorical predictor, and the log of the population denominator as an offset in the models for rates. We then evaluated heterogeneity and trend across years using Wald tests, based on the fitted models. To assess the independent associations of age, sex, race/ethnicity, median income, insurance, ISS, mechanism of injury, and trauma center care with numbers of revisits and readmissions within 1 year, we used multivariate negative binomial models, again with robust standard errors to account for clustering by hospital. To account for the competing risk of death, these models used the log of the follow-up time as an offset, with appropriately shorter follow-up for patients who died. Finally, to identify risk factors associated with 1-year mortality, both overall and for discharged and admitted patients, we used analogous multivariate logistic models to calculate hazard ratios (HR), again with robust standard errors to account for clustering by hospital. All regressions were performed for TBI and Other Trauma separately. We conducted all analyses using SAS (version 9.2; Cary, NC). The University of California, San Francisco Institutional Review Board approved our study and issued a waiver for consent for using non-anonymized data.

## Results

We identified 208,222 patients aged ≤17 years with an index diagnosis of TBI during their ED evaluation and 1,314,064 patients with an index diagnosis of Other Trauma (Table 1). Compared with Other Trauma patients, TBI patients were, on average, younger (mean of 8.1 vs. 9.5 years, p<0.001), more likely to be male (62.7% vs. 60.4%, p<0.001), privately insured (46.3% vs. 43.3%, p<0.001), and more severely injured (mean ISS of 4.8 vs. 4.5, p<0.001) (Table 1). Compared with Other Trauma patients, mechanisms of injury for TBI patients were more commonly falls (48.3% vs. 33.5%, p<0.001) and motor vehicle crashes (6.8% vs. 3.7%, p<0.001), they were more likely to be treated at a trauma center (28.7% vs. 24.7%, p<0.001), and were more likely to be admitted as an inpatient (6.8% vs. 3.5%, p<0.001).

**Table 1:** Summary of patient characteristics of pediatric index visits: TBI vs. other trauma.

	Other Trauma (N = 1,314,064)		TBI (N = 208,222)		
	N (%)	Mean (SD)	N (%)	Mean (SD)	p-value
*Age (years)*		10 (5–14)		8 (2–14)	<0.001
0–4	310123 (23.6)		77356 (37.2)		
5–9	302906 (23.1)		40497 (19.5)		
10–14	397832 (30.3)		45497 (21.9)		
15–17	303203 (23.1)		44872 (21.6)		
*Sex*					<0.001
Male	794189 (60.4)		130600 (62.7)		
Female	519137 (39.5)		77571 (37.3)		
Missing	738 (0.1)		51 (0)		
*Race/Ethnicity*					<0.001
Non-Hispanic White	432300 (32.9)		70370 (33.8)		
Non-Hispanic Black	138121 (10.5)		21400 (10.3)		
Hispanic	574312 (43.7)		88675 (42.6)		
Other	127284 (9.7)		21025 (10.1)		
Missing	42047 (3.2)		6752 (3.2)		
Median income		54329 (44069–68447)		56507 (45535–71775)	<0.001
*Insurance*					<0.001
Private	569302 (43.3)		96467 (46.3)		
Medicare	7392 (0.6)		1248 (0.6)		
Medicaid	567060 (43.2)		85706 (41.2)		
Self-pay/Uninsured	110223 (8.4)		15189 (7.3)		
Other	60087 (4.6)		9612 (4.6)		
*Injury Severity Score*		1 (1–4)		4 (4–5)	<0.001
<9	1264432 (96.2)		196572 (94.4)		
9–15	14216 (1.1)		7590 (3.7)		
≥16	35416 (2.7)		4060 (2)		
*Injury characteristics (E-code)*					<0.001
Penetrating Injury	122747 (9.3)		926 (0.4)		
Falls	440645 (33.5)		100623 (48.3)		
Motor Vehicle Crash	48488 (3.7)		14221 (6.8)		
Other	504435 (38.4)		73581 (35.3)		
Missing	197749 (15.1)		18871 (9.1)		
*Received care at level I or II Trauma Center*					<0.001
No	989244 (75.3)		148519 (71.3)		
Yes	324820 (24.7)		59703 (28.7)		
*Disposition of index visit*					<0.001
Discharged (or died in) from ED	1267942 (96.5)		193999 (93.2)		
Admitted to hospital	46122 (3.5)		14223 (6.8)		
*Died in ED*					<0.001
No	870914 (100)		122100 (100)		
Yes	139 (0)		66 (0.1)		
Length of stay if admitted		2 (1–4)		2 (1–4)	<0.001
*Died in Hospital (Inpatient)*					<0.001
No	33058 (99.7)		9977 (97.9)		
Yes	100 (0.3)		217 (2.1)		

Discharged or died in ED includes discharged or transferred to home under care of a Home Intravenous provider from the ED for years 2005 and 2006 only. Percentages may not add up to 100% due to rounding error.

Abbreviations: ED—emergency department; TBI—traumatic brain injury; SD—standard deviation

**Fig 1:**
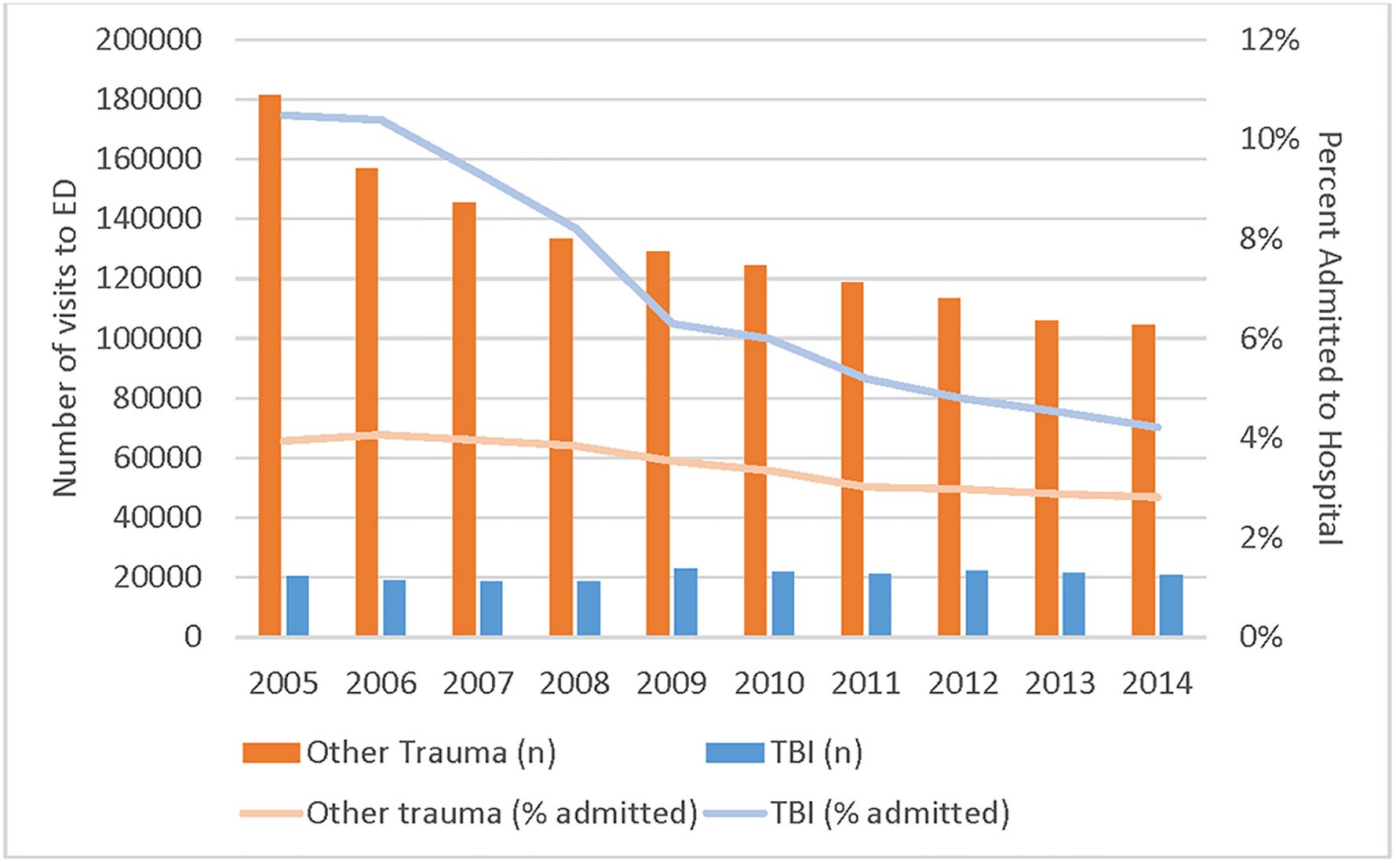
Number of visits and percent of visits admitted to hospital for pediatric TBI and other trauma.

Visits with a TBI diagnosis in absolute numbers increased by 2.7% while those for Other Trauma decreased by 42.4% from 2005 to 2014 (S1 Table). However, Fig 1 shows that the percent of visits for pediatric TBI resulting in hospitalization declined from 10.5% to 4.2% over the study period, with a relatively stable proportion of Other Trauma visits that were admitted (3.9% in 2005 and 2.8% in 2014).

When accounting for population growth, ED visit rates for TBI (Table 2) increased by 5.6% during the study period, while Other Trauma visits decreased by 40.7% (p<0.001). TBI visit rates most notably increased in patients who were female (+15.7%, p<0.001) age <5 years (+25.0%, p<0.001), non-Hispanic black (+22.2%, p<0.001), Hispanic (+24.7%, p<0.001), Other Race/Ethnicity (+21.6%, p<0.001), privately insured (+18.5%, p<0.001), and uninsured (+79.5%, p<0.001) patients. In contrast, Other Trauma visit rates markedly decreased across all demographics (range of 28.9% to 68.7%, p<0.001) except in the uninsured who showed a slight increase.

**Table 2:** Pediatric emergency department index visit rates (per 100,000 residents) from 2005–2014: TBI vs. other trauma^a^.

		2005	2014	p-value (trend)
**Total**	Other trauma	1930.35	1145	<0.001
	TBI	217.46	229.7	<0.001
**Sex**				
* Male*	Other trauma	2343.95	1350.03	<0.001
	TBI	275.47	281.56	<0.001
* Female*	Other trauma	1481.32	948.48	<0.001
	TBI	155.71	179.96	<0.001
**Age (years)**				
*< 5*	Other trauma	1535.05	1056.79	<0.001
	TBI	270.66	338.32	<0.001
* 5–9*	Other trauma	1714.98	991.46	<0.001
	TBI	168.03	164.8	0.12
* 10–14*	Other trauma	2047.69	1225.24	<0.001
	TBI	164.18	170.15	<0.001
* 15–17*	Other trauma	2662.94	1408.64	<0.001
	TBI	297.46	258.16	<0.001
**Race/Ethnicity**				
* Non-Hispanic White*	Other trauma	2252.88	1286.21	<0.001
	TBI	263.21	262.8	<0.001
* Non-Hispanic Black*	Other trauma	3127.77	2159.21	<0.001
	TBI	350.31	428.16	<0.001
* Hispanic*	Other trauma	1558.01	1051.41	<0.001
	TBI	166	207.05	<0.001
* Other*	Other trauma	1464.43	1040.54	<0.001
	TBI	177.15	215.37	<0.001
**Insurance**				
* Private*	Other trauma	1295.02	836.15	<0.001
	TBI	152.59	180.75	<0.001
* Medicaid*	Other trauma	3085.81	1536.28	<0.001
	TBI	328.74	289.01	<0.001
* Uninsured*	Other trauma	1221.25	1229.38	<0.001
	TBI	135.84	243.86	<0.001
* Other Insured*	Other trauma	5433.54	1698.09	<0.001
	TBI	655.58	371.34	<0.001

^a^Residents refer to the population within the specified demographic group in California for that year.

For example, residents for male is derived from the male pediatric (ages 0–17) population in California.

Abbreviations: TBI—traumatic brain injury

### Discharge dispositions

The majority of pediatric patients with a TBI or Other Trauma index visit were discharged from the ED (93.2% versus 96.5%, p<0.001, Table 1). The proportion of patients admitted to the hospital (inpatient) decreased for both TBI and Other Trauma; however, TBI patients experienced a larger decrease, from 10.5% in 2005 to 4.2% in 2014 (vs. 4.0% to 2.8% for Other Trauma, p<0.001) (S1 Table).

Overall, we found that the utilization of other and longer-term healthcare services increased during the study period, both from the ED and inpatient settings (S2 Table). Although the absolute numbers are small, the proportion of patients discharged directly from the ED after a TBI index visit to “other healthcare institution” (such as federal hospital or critical access hospital) increased nearly 10-fold from 2005 to 2014 (0.05% to 0.46%, p<0.001). We observed similar trends for dispositions of Other Trauma index visits. Of patients discharged from inpatient to elsewhere, some TBI patients (10.7%) but disproportionately more Other Trauma patients were discharged with home health service (32.2%, p<0.001) (S1 Fig).

### Revisits, readmissions, and inpatient charges

A total of 512,646 ED revisits and 45,182 readmissions occurred after an index TBI visit, which is an average of 2.5 ED revisits per TBI patient (vs. 0.4 for Other Trauma, p<0.001) and 1 readmission per 5 TBI patients (vs. 1 per 30 for Other Trauma, p<0.001) (Table 3). Of all index TBI who had a readmission within 1 year, 70.9% (95% CI 68.8%– 72.9%) had 1 readmission and 7.8% had ≥ 4 readmissions within that year (95% CI 6.7%–9.1%). For all index Other Trauma patients with at least 1 readmission, 62.8% (95% CI 58.3%–67.3%) had 1 readmission and 9.4% (95% CI 6.8%–12.4%) had ≥ 4 readmissions within that year (Table 3).

**Table 3:** Revisits and readmissions (any type of visit not restricted to TBI/other trauma) of pediatric index visits: TBI vs. other trauma.

	Other trauma (N = 1,314,064)						TBI (N = 208,222)						
	N (%)	Mean	SD	Median	Q1	Q3	N (%)	Mean	SD	Median	Q1	Q3	P-value
Total revisits	522565						512646						< .0001
Revisits within 1 year of index visit	78721 (15.1)						127553 (24.9)						< .0001
*Patients with ≥1 revisit within 1 year of index visit*	38700 (3)						69497 (33.4)						< .0001
1 revisit	20368 (52.6)						41176 (59.3)						
2 revisits	9364 (24.2)						15089 (21.7)						
3 revisits	4219 (10.9)						6417 (9.2)						
4 revisits	2110 (5.5)						3130 (4.5)						
5 to 9 revisits	2320 (6)						3327 (4.8)						
≥10 revisits	319 (0.8)						358 (0.5)						
Days from discharge to revisit		171.44	109.65	169	74	266		163.24	108.94	156	66	256	< .0001
*Revisited the same hospital as index visit*?													< .0001
Yes	60105 (76.4)						99331 (77.9)						
No	18616 (23.7)						28222 (22.1)						
*Primary Diagnosis at revisit*													< .0001
TBI	14772 (18.8)						6062 (4.8)						
non-TBI	63949 (81.2)						121491 (95.3)						
**Hospital Readmissions**													
Total readmissions	44517						45182						< .0001
Readmissions within 1 year of index visit	887 (2)						3181 (7)						< .0001
*Patients with ≥1 readmission within 1 year of index visit*	460 (0)						1900 (0.9)						< .0001
1 readmission	289 (62.8)						1347 (70.9)						
2 readmissions	96 (20.9)						308 (16.2)						
3 readmissions	32 (7)						96 (5.1)						
4 readmissions	11 (2.4)						51 (2.7)						
5 to 9 readmissions	24 (5.2)						81 (4.3)						
≥10 readmissions	8 (1.7)						17 (0.9)						
Days from discharge to all readmission		134.45	112.86	113	28	223		97.34	110.65	50	1	169	< .0001
*Readmitted to the same hospital as index visit*?													< .0001
Yes	424 (47.8)						1795 (56.4)						
No	463 (52.2)						1386 (43.6)						
*Type of readmission*													< .0001
Scheduled	166 (18.7)						1190 (37.4)						
Unscheduled	721 (81.3)						1990 (62.6)						
Other							1						
*Primary Diagnosis at readmission*													< .0001
TBI	78 (8.8)						463 (14.6)						
non-TBI	809 (91.2)						2718 (85.4)						
**Mortality**													
Total deaths from 2005–2010	1007 (0.1)						422 (0.2)						< .0001
Median time to death		590.91	569.18	442	38	1018		227.28	430.65	3	0	262	< .0001
*Died within…*?													< .0001
Index hospitalization	308 (0.0)						371 (0.2)						
30 days after index visit	30 (0.0)						14 (0.0)						
31 to 60 days after index visit	20 (0.0)						4 (0.0)						
61days to a year after index visit	192 (0.0)						36 (0.0)						
**Costs**													
Inpatient costs incurred within 1 year of index visit		40279.92	115068.76	18694.50	7942	37850		66574.50	154347.49	24750.00	9267	56915	< .0001

Notes: Mortality only includes patients with vital statistics data, and the data covers up until 2011, the most currently available year from the California Office of Statewide Health Planning and Development. Percentages may not add up to 100% due to rounding error.

Abbreviations: TBI—traumatic brain injury; SD—standard deviation; ED—emergency department

We found that 33.4% (95% CI 33.2%–33.6%) of all patients with an index TBI visit had at least 1 ED revisit and 0.9% (95% CI 0.87%–0.95%) had at least 1 hospital readmission within the first year, comprising 127,553 revisits and 3,181 readmissions during the study period. The median time from discharge to an ED revisit was 156 days (interquartile range [IQR] 66, 256), with 8.9% of revisits or readmissions for TBI patients occurring within 14 days of the index visit. The pattern of revisits was markedly different for Other Trauma patients, with only 3.0% (95% CI 2.91% -2.97%) of patients revisiting the ED and 0.04% (95% CI 0.032% -0.038%) of patients readmitted during the first year after discharge. The median time from discharge to revisit was 169 days (IQR 74, 266), with 8.4% of revisits or readmissions occurring within 14 days of the index visit for Other Trauma patients. Notably, 18.8% (95% CI 18.5%–19.0%) of ED revisits for Other Trauma patients resulted in a TBI diagnosis (vs. 4.8% for TBI patients, (95% CI 4.6%–4.9%), while 14.6% (95% CI 13.3%–15.8%) of readmissions for TBI patients resulted in a TBI diagnosis (vs. 8.8% (95% CI 7.0%–10.9%) for Other Trauma patients).

**Fig 2:**
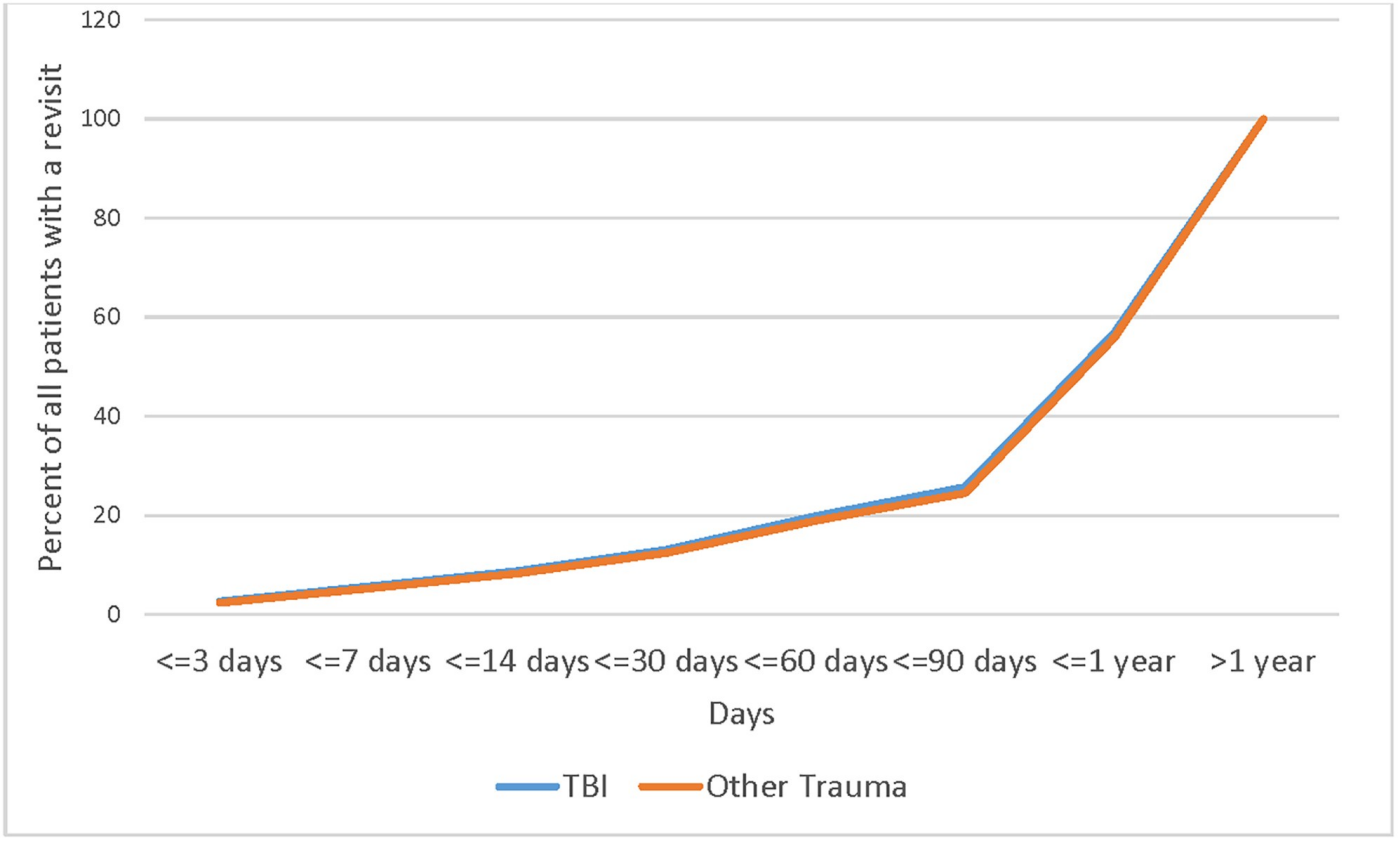
Time from discharge to first revisit for pediatric TBI and other trauma.

However, despite the significantly larger proportion of pediatric patients who were diagnosed with TBI on the first visit that had at least 1 ED visit compared to the non-TBI Other Trauma cohort, of those who did have an ED revisit, the timing of these revisits were remarkably similar. Fig 2 shows that the proportion of patients with revisits at varying timepoints is nearly identical.

Risk factors associated with revisits and readmissions after both index TBI visits and Other Trauma visits included older age, female sex, race, insurance, and injury severity and mechanism (S3 Table). The median inpatient charge for a hospital admission with TBI was approximately $6000 greater than that for an Other Trauma admission ($24,750 vs. $18,695, p<0.001) (Table 3).

### Mortality

Compared with Other Trauma patients, TBI patients were more than twice as likely to die in the ED (0.05% vs. 0.02%, p<0.001) and more than 7 times as likely to die in the hospital (inpatient) (2.1% vs. 0.3%, p<0.001) (Table 1). Additionally, index TBI patients had more than a 3-fold greater risk of mortality within 30 days of the index visit (0.007% vs. 0.002%, p<0.001) and were over 6 times more likely to die within 1 year of the index TBI visit than Other Trauma patients (0.2% vs. 0.04%, p<0.001) (Table 3).

Mortality risk factors for TBI patients differed considerably from those for Other Trauma patients (Table 4). For TBI patients, patients aged 0–4 years had a 2.1 (95% CI 1.6 to 2.9) times greater likelihood of mortality than the oldest patient group (15–17 years) while the reverse trend was seen for Other Trauma patients. Underinsured patients had a higher risk of mortality: uninsured TBI patients and Medicaid-insured Other Trauma patients had higher likelihoods of mortality (hazard ratio (HR) 1.9, 95% CI 1.2 to 2.9 for TBI; RH 1.3, 95% CI 1.1 to 1.6 for Other Trauma) relative to privately insured TBI and Other Trauma patients. Penetrating injuries were associated with higher likelihood of mortality for both TBI and Other Trauma (HR 4.1, 95% CI 2.9 to 5.7; and HR 1.4, 95% CI 1.0 to 2.0, respectively), while falls and other injury mechanisms were associated with lower likelihood of mortality, relative to motor vehicle crash injuries.

**Table 4:** Multivariate regression results of odds of death within 1 year of discharge: Pediatric TBI vs. other trauma patients.

	All				Discharged from the ED				Admitted to the hospital			
	Other Trauma		TBI		Other Trauma		TBI		Other Trauma		TBI	
	RH	95% CI	RH	95% CI	RH	95% CI	RH	95% CI	RH	95% CI	RH	95% CI
*Age (ref*. *grp*.: *15–17 years)*												
0–4 years	0.63**	0.49–0.80	2.12**	1.57–2.87	0.52**	0.38–0.71	3.30**	1.81–6.01	1.13	0.73–1.73	1.62**	1.13–2.31
5–9 years	0.41**	0.30–0.55	1.18	0.82–1.71	0.28**	0.19–0.42	1.47	0.73–2.97	1.00	0.62–1.61	1.01	0.65–1.59
10–14 years	0.50**	0.39–0.64	1.01	0.73–1.39	0.37**	0.27–0.51	1.30	0.67–2.54	1.06	0.70–1.59	0.89	0.62–1.30
*Sex (ref*. *grp*.: *Female)*												
Male	1.28*	1.05–1.56	1.13	0.89–1.44	1.44**	1.12–1.86	0.91	0.58–1.42	1.08	0.78–1.48	1.19	0.89–1.59
Missing	3.07	0.74–12.73	0.00	0.00–.	4.57*	1.06–19.62	0.00	0.00–.	0.00	0.00–.	0.00	0.00–.
*Race/Ethnicity (ref*. *grp*.: *Non-Hispanic White)*												
Non-Hispanic Black	1.61**	1.19–2.19	1.36	0.93–1.99	1.71**	1.18–2.47	1.19	0.53–2.70	1.63+	0.95–2.81	1.42	0.92–2.19
Hispanic	1.39**	1.09–1.78	1.09	0.81–1.45	1.20	0.88–1.63	1.22	0.69–2.15	1.76**	1.17–2.66	0.98	0.70–1.38
Other	1.62**	1.17–2.25	1.27	0.86–1.87	1.32	0.86–2.03	1.11	0.50–2.45	2.15**	1.29–3.60	1.16	0.74–1.82
Missing	1.90**	1.19–3.04	1.84+	0.95–3.57	2.39**	1.44–3.98	1.14	0.41–3.18	1.40	0.34–5.84	2.04	0.82–5.11
Median income (per $1000)	1.00	1.00–1.01	0.99**	0.98–1.00	1.00	0.99–1.01	0.99+	0.98–1.00	1.00	1.00–1.01	0.99*	0.98–1.00
*Insurance (ref*. *grp*.: *private insurance)*												
Medicare	1.26	0.31–5.09	0.00	0.00–2E242	1.75	0.43–7.14	0.00	0.00–.	0.00	0.00–.	0.00	0.00–3E250
Medicaid	1.31*	1.05–1.64	1.06	0.79–1.41	1.48**	1.11–1.98	1.34	0.79–2.25	1.01	0.71–1.45	1.01	0.72–1.42
Self-pay/Uninsured	1.06	0.75–1.50	1.88**	1.21–2.93	1.44+	0.97–2.14	1.66	0.85–3.23	0.61	0.22–1.70	1.59	0.84–2.98
Other	1.17	0.82–1.68	1.10	0.76–1.60	1.07	0.63–1.82	1.94	0.87–4.31	0.89	0.54–1.48	1.06	0.69–1.63
*Injury Severity Scores (ref*. *grp*.: *< 9)*												
9–15	8.24**	6.25–10.87	14.52**	9.65–21.86	44.41**	31.71–62.20	6.30**	2.64–15.07	0.87	0.56–1.34	5.40**	2.63–11.07
≥ 16	10.25**	7.86–13.37	76.09**	53.33–108.6	6.43**	3.94–10.50	182.9**	113.1–295.8	3.95**	2.76–5.65	22.05**	11.19–43.45
*Injury characteristics (E- codes) (ref*. *grp*.: *Any Motor Vehicle Crash)*												
Penetrating Injury	1.40*	1.00–1.96	4.06**	2.89–5.69	1.11	0.75–1.65	2.16*	1.02–4.57	3.34**	1.73–6.44	4.97**	3.36–7.35
Falls	0.28**	0.19–0.42	0.21**	0.14–0.32	0.19**	0.12–0.31	0.04**	0.02–0.08	0.58	0.27–1.27	0.46**	0.29–0.74
Other	0.27**	0.19–0.38	0.33**	0.22–0.48	0.18**	0.12–0.28	0.10**	0.05–0.19	1.07	0.54–2.09	0.55	0.35–0.88
Missing	1.07	0.76–1.51	0.65**	0.47–0.89	0.42**	0.27–0.64	0.17**	0.09–0.35	3.68**	1.97–6.87	1.03	0.72–1.49
*Received care at level I or II trauma center (ref*. *grp*.: *did not receive care at level I or II trauma center)*												
Yes	2.77**	2.28–3.37	1.24	0.94–1.64	2.77**	2.17–3.52	1.28	0.82–2.01	1.20	0.87–1.67	1.32	0.93–1.88

Abbreviations: TBI—traumatic brain injury; CI—confidence interval; RH—relative hazard.

^+^ p<0.10

* p<0.05

**p<0.01

## Discussion

We evaluated over 1.5 million pediatric trauma patient visits to California EDs over a 10-year period and found a dramatic difference in ED visit trends for patients with TBI versus Other Trauma. Population growth adjusted ED visits with a TBI diagnosis increased by 5.6% while those for Other Trauma decreased by 40.7%. ED pediatric TBI visits nadired in 2007 and peaked in 2012, resulting in an almost 20% increase in that time period, similar to trends reported in the most recent CDC report on the national incidence of TBI from 2007 to 2013[1] and the equivalent adult TBI study in California.[27] The overall increase in ED visits for pediatric TBI are consistent with other studies that implicate state and national public health initiatives that have increased awareness, and therefore, likelihood of diagnosis. An increase in the likelihood of TBI diagnosis (as opposed to true incidence) could also partially explain the dramatic decrease in Other Trauma, as these groups are mutually exclusive.[28] The rate of visits to the ED for TBI increased significantly for certain groups, including females, children under 5, and minorities (Blacks, Hispanics, and Other). Varying trends across differently insured patients also emerged as an important finding, suggesting that these increases in TBI visits are not only a condition of patients with less socioeconomic advantage: TBI visits to the ED noticeably increased in uninsured pediatric patients, with a less dramatic but still substantial increase in privately insured patients, but Medicaid-insured patients experienced a decrease in the rate of visits to the ED for TBI.

We also found important differences between TBI and Other Trauma patients in ED revisits and readmissions. Prior estimates of ED revisits are limited to shorter time frames, e.g., 72 hours after injury,[29] which may not fully capture longer-term healthcare needs. We found that one third of TBI patients had at least one ED revisit within a year of the index visit, compared with only 3.0% for Other Trauma patients, and that the proportion of TBI patients readmitted within a year of discharge was more than twice the proportion of readmitted Other Trauma patients. In addition, a non-trivial 0.2% of patients died within a year of an index TBI visit, representing a 5-fold increased risk compared with Other Trauma patients. These findings suggest that pediatric TBI is associated with a post-injury disease burden that exceeds that of nonspecific trauma. Moreover, the ED revisit rates are comparable to those described in children with chronic diseases such as asthma,[30] suggesting that TBI is not a one-time event but rather, may need to be treated in a fashion similar to chronic conditions. While our study design cannot determine if the index TBI injury is the definitive cause of the revisits, other studies suggest that TBI can result in a host of adverse sequelae (ranging from gastrointestinal to neuropsychiatric),[14,15] delayed symptoms, and other longer-term adverse outcomes.[4–9]

Overall, our findings suggest that a system of follow-up care may not only better meet the healthcare needs of pediatric patients with TBI but also more efficiently allocate healthcare resources and decrease unscheduled return visits to the ED, reducing overall healthcare costs and ED overcrowding.[10–13,31,32] Findings from another study evaluating outpatient-only utilization for head injury, with an average of 2.5 visits post-index ambulatory ED visit for TBI within 30 days. Providing outpatient healthcare and home services, including psychosocial and emotional support, as well as early identification and treatment of TBI sequelae, may lessen the burden of parents and guardians, while improving pediatric TBI recovery.[33] Further studies evaluating high-risk clinical features could better inform and support the use of outpatient healthcare services in the pediatric TBI population.

### Limitations

This study has several limitations. First, our study is clearly a conservative estimate and most certainly underestimates the complete number of pediatric injuries, as a large number of encounters may occur in urgent care and primary care provider settings.[3] Second, our dataset contains a limited amount of clinical information and does not include objective measures of neurologic status such as the Glasgow Coma Score (GCS) or advanced imaging results. Therefore, as with previous studies, we used ISS to classify the general severity of the overall diagnosis.[23,24] Third, the construction of the study cohorts could be confounded by injury severity, which in turn would influence the study outcomes. The cohort of TBI patients was constructed utilizing any patient with TBI, including that of multisystem trauma. As one might suspect, we found that the ISS was significantly greater in those pediatric patients with TBI compared with those with Other Trauma, 4.8 vs. 4.5, and there was a higher percentage of moderately injured patients with TBI compared with non-TBI trauma patients (3.7% vs. 1.1%). However, we are unsure of the clinical relevance of this difference in ISS, since the ISS score is valued from 0–75. Moreover, and unexpectedly, we found that TBI patients constituted a smaller proportion of severely injured patients (ISS ≥ 16) than that of the Other Trauma patients, 2.0% vs. 2.7%. These findings suggest that our study could underrepresent the difference in the rate of revisit and mortality in patients with TBI compared with those with Other Trauma. Finally, it is difficult to determine the relationship between revisits and readmissions to the initial injury. We felt it was important to capture all revisits and readmissions, since many diagnoses may be attributable to the original TBI but be diagnosed with a different name–for example, someone feeling dizzy or having headaches presenting for care could be coded with simply the symptoms. But certainly there are many instances where the revisit or readmission may not have anything to do with the index injury. For these reasons, we provide the non-TBI trauma as a comparison. Even then, however, there is no way to definitively determine the relationship between revisits or readmissions with the initial injury.

## Conclusions

The number of pediatric TBI-related ED visits in California increased at the same time that visits for Other Trauma markedly decreased. TBI but not Other Trauma patients had revisit rates similar to those reported in children with chronic diseases. The 1-year health burden after a pediatric TBI visit is considerable, and outpaces that of nonspecific trauma. Overall, our findings suggest that TBI patients fare differently from Other Trauma patients and may have greater, unmet longer-term healthcare needs. Our healthcare system should move toward more efficient and effective post-discharge care of pediatric TBI, with strategies to monitor for longer-term sequelae to help improve patient outcomes, lessen the burden on families, and more appropriately and efficiently allocate healthcare resources.

## Supporting information

S1 Fig Disposition of pediatric index visits: Inpatient hospitalizations discharged to elsewhere (not home). Notes: Other type of hospital care includes psychiatric, chemical dependency, physical medicine rehabilitation. Abbreviations: TBI—traumatic brain injury; ED—emergency department; SNF—skilled nursing facility.
(TIFF)

S1 Methods Population data, linkage of encounters, hospital-level variables, and identification of TBI and other trauma patients. (DOCX)

S1 References (DOCX)

S1 Table Summary of patient characteristics by year from 2005–2014: Pediatric TBI vs. other trauma index visits. ^a^Percentages may not add to 100% due to rounding error. Empty cells are due to lack of death data or lack of any observations for the specified category and year. ^b^Discharged or died in ED includes discharged or transferred to home under care of a Home Intravenous provider from the ED for years 2005 and 2006 only. Abbreviations: ED—emergency department; SD—standard deviation; TBI—traumatic brain injury; TC—trauma center.
(DOCX)

S2 Table Disposition and outcomes by year from 2005–2014: Pediatric TBI vs. other trauma index visits. ^*a*^Percentages may not add to 100% due to rounding error. Empty cells are due to lack of death data or lack of any observations for the specified category and year. ^*b*^e.g. federal, critical access hospital, psychiatric, cancer. ^*c*^e.g. psychiatric, chemical dependency, physical medicine rehabilitation. Abbreviations: TBI—traumatic brain injury; ED—emergency department; SNF—skilled nursing facility; IRF—intermediate rehabilitation facility; LTCH—long-term care hospital.
(DOCX)

S3 Table Negative binomial regression results for revisit and readmissions: Pediatric TBI vs. other trauma patients. Abbreviations: TBI—traumatic brain injury; CI—confidence interval; RH—relative hazard. + p<0.10 * p<0.05 ** p<0.01.
(DOCX)
