# Supplementary figures and images for: Revisits, readmissions, and outcomes for pediatric traumatic brain injury in California, 2005-2014
Source: PLoS One. 2020 Jan 24;15(1):e0227981. doi: 10.1371/journal.pone.0227981 (PMC6980591; doi:10.1371/journal.pone.0227981)

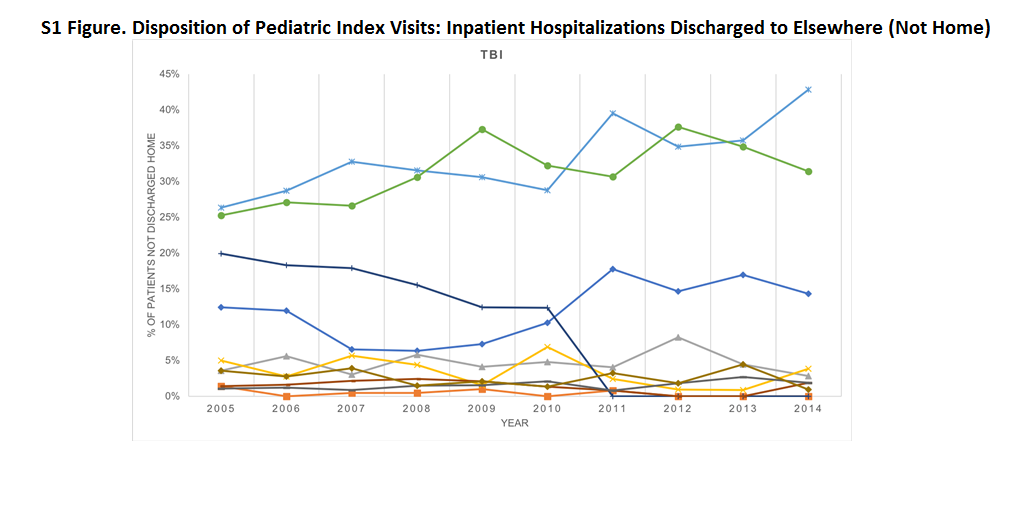

Supplement: S1 Fig — Notes: Other type of hospital care includes psychiatric, chemical dependency, physical medicine rehabilitation. Abbreviations: TBI—traumatic brain injury; ED—emergency department; SNF—skilled nursing facility. (TIFF) [file pone.0227981.s001.tiff]
