# Supplementary material for: Revisits, readmissions, and outcomes for pediatric traumatic brain injury in California, 2005-2014
Source: PLoS One. 2020 Jan 24;15(1):e0227981. doi: 10.1371/journal.pone.0227981 (PMC6980591; doi:10.1371/journal.pone.0227981)
Supplement: S1 Methods — (DOCX) [file pone.0227981.s002.docx]

**S1 Methods. Population Data, Race/Ethnicity Classification, Linkage of Encounters, Hospital-Level Variables, and Identification of TBI and Other Trauma Patients**

**Population Data**

To calculate pediatric TBI and Other Trauma visit rates by race, age, sex, and insurance type, we obtained California sex and age population counts from the US Census and race/ethnicity population counts from the State of California, Department of Finance (2005-2009) and the American Community Survey (ACS) (2010-2014). We calculated visit rates by payer using population insurance information from the Current Population Survey (CPS) for 2005-2012 and the ACS for 2013-2014.

**Race/Ethnicity Classification**

OHSPD collects race/ethnicity data from hospitals, which are self-reported. A patient’s ethnicity can be classified as Hispanic, non-Hispanic, or Unknown. A patient’s race can be classified as white, black, Native American/Eskimo/Aleut, Asian/Pacific Islander, Other, and Unknown. We used a normalized race variable provided by OSHPD, which categorizes patients as Hispanic if the patient reported Hispanic ethnicity, and indicates the patient’s race otherwise. We examined race/ethnicity because there may be underlying differences in TBI incidence among the different racial/ethnic groups.

**Hospital-Level Variables**

In California, the local Emergency Medical Services (EMS) agency designates hospitals as trauma centers according to their own criteria and reports this information in the OSHPD hospital data files,^1^ which is then used to rank all hospitals from Level I to IV. We defined Level I and II hospitals as trauma centers, and Level III and IV hospitals as non-trauma centers, consistent with the current literature.^2,3^
