# Supplementary material for: Revisits, readmissions, and outcomes for pediatric traumatic brain injury in California, 2005-2014
Source: PLoS One. 2020 Jan 24;15(1):e0227981. doi: 10.1371/journal.pone.0227981 (PMC6980591; doi:10.1371/journal.pone.0227981)
Supplement: S1 References — (DOCX) [file pone.0227981.s003.docx]

**S1 References.**

1. California Office of Statewide Health Planning and Development - Healthcare Information Division. Hospital Annual Utilization Data Pivot Profiles. https://www.oshpd.ca.gov/HID/Hospital-Utilization.html. Accessed August 2, 2018.

2. Newgard CD, Staudenmayer K, Hsia RY, Mann NC, Bulger EM, Holmes JF, et al. The cost of overtriage: More than one-third of low-risk injured patients were taken to major trauma centers. *Health Aff*. 2013;32(9):1591-1599.

3. Hsia RY, Wang E, Saynina O, Wise P, Pérez-Stable EJ, Auerbach A. Factors associated with trauma center use for elderly patients with trauma: a statewide analysis, 1999-2008. *Arch Surg*. 2011;146(5):585-592.
