# Supplementary material for: Revisits, readmissions, and outcomes for pediatric traumatic brain injury in California, 2005-2014
Source: PLoS One. 2020 Jan 24;15(1):e0227981. doi: 10.1371/journal.pone.0227981 (PMC6980591; doi:10.1371/journal.pone.0227981)
Supplement: S1 Table — aPercentages may not add to 100% due to rounding error. Empty cells are due to lack of death data or lack of any observations for the specified category and year. bDischarged or died in ED includes discharged or transferred to home under care of a Home Intravenous provider from the ED for years 2005 and 2006 only. Abbreviations: ED—emergency department; SD—standard deviation; TBI—traumatic brain injury; TC—trauma center. (DOCX) [file pone.0227981.s004.docx]

**S1 Table. Summary of Patient Characteristics by Year from 2005-2014: Pediatric TBI vs. Other Trauma Index Visits**

|  |  | **2005** | **2006** | **2007** | **2008** | **2009** | **2010** | **2011** | **2012** | **2013** | **2014** | **p-value (trend)** | |
| --- | --- | --- | --- | --- | --- | --- | --- | --- | --- | --- | --- | --- | --- |
|  |  | N (%) | N (%) | N (%) | N (%) | N (%) | N (%) | N (%) | N (%) | N (%) | N (%) | Other trauma | TBI |
| **Total** | Other trauma | 181560 | 156991 | 145391 | 133388 | 129155 | 124568 | 118642 | 113593 | 106110 | 104666 |  |  |
|  | TBI | 20453 | 19136 | 18858 | 18806 | 22969 | 21944 | 21122 | 22422 | 21515 | 20997 |  |  |
| **Sex** |  |  |  |  |  |  |  |  |  |  |  | <0.001 | <0.001 |
| *Male* | Other trauma | 112907  (62.2) | 97530  (62.1) | 90421  (62.2) | 81853  (61.4) | 78096  (60.5) | 74718  (60.0) | 70299  (59.3) | 66391  (58.5) | 61588  (58.0) | 60386  (57.7) |  |  |
|  | TBI | 13269  (64.9) | 12269  (64.1) | 12145  (64.4) | 11979  (63.7) | 14525  (63.2) | 13738  (62.6) | 13170  (62.4) | 13745  (61.3) | 13166  (61.2) | 12594  (60.0) |  |  |
| *Female* | Other trauma | 67972  (37.4) | 59443  (37.9) | 54955  (37.8) | 51533  (38.6) | 51052  (39.5) | 49849  (40.0) | 48341  (40.8) | 47193  (41.6) | 44522  (42.0) | 44277  (42.3) |  |  |
|  | TBI | 7145  (34.9) | 6865  (35.9) | 6709  (35.6) | 6826  (36.3) | 8443  (36.8) | 8206  (37.4) | 7952  (37.7) | 8677  (38.7) | 8347  (38.8) | 8401  (40.0) |  |  |
| *Missing* | Other trauma | 681  (0.4) | 18  (0.0) | 15  (0.0) | 2  (0.0) | 7  (0.0) | 1  (0.0) | 2  (0.0) | 9  (0.0) | - | 3  (0.0) |  |  |
|  |  |  |  |  |  |  |  |  |  | - |  |  |  |
|  | TBI | 39  (0.2) | 2  (0.0) | 4  (0.0) | 1  (0.0) | 1  (0.0) | -  - | -  - | -  - | 2  (0.0) | 2  (0.0) |  |  |
| **Age (years)** |  |  |  |  |  |  |  |  |  |  |  | <0.001 | <0.001 |
| *0 – 4* | Other trauma | 39230  (21.6) | 33480  (21.3) | 31707  (21.8) | 30966  (23.2) | 31807  (24.6) | 30876  (24.8) | 30197  (25.5) | 28786  (25.3) | 26617  (25.1) | 26457  (25.3) |  |  |
|  | TBI | 6917  (33.8) | 6420  (33.6) | 6323  (33.5) | 6872  (36.5) | 8668  (37.7) | 8458  (38.5) | 8238  (39.0) | 8738  (39.0) | 8252  (38.4) | 8470  (40.3) |  |  |
| *5 – 9* | Other trauma | 41621  (22.9) | 36213  (23.1) | 33612  (23.1) | 30388  (22.8) | 28878  (22.4) | 28138  (22.6) | 27081  (22.8) | 26426  (23.3) | 25221  (23.8) | 25328  (24.2) |  |  |
|  | TBI | 4078  (19.9) | 3778  (19.7) | 3730  (19.8) | 3648  (19.4) | 4505  (19.6) | 4214  (19.2) | 3991  (18.9) | 4152  (18.5) | 4191  (19.5) | 4210  (20.1) |  |  |
| *10 – 14* | Other trauma | 56825  (31.3) | 48654  (31.0) | 44059  (30.3) | 40035  (30.0) | 38814  (30.1) | 37658  (30.2) | 35396  (29.8) | 33912  (29.9) | 31522  (29.7) | 30957  (29.6) |  |  |
|  | TBI | 4556  (22.3) | 4264  (22.3) | 4184  (22.2) | 3953  (21.0) | 5055  (22.0) | 4741  (21.6) | 4483  (21.2) | 5057  (22.6) | 4905  (22.8) | 4299  (20.5) |  |  |
| **S1 Table. Summary of Patient Characteristics by Year from 2005-2014: Pediatric TBI vs. Other Trauma Index Visits (continued)** | | | | | | | | | | | | | |
|  |  | **2005** | **2006** | **2007** | **2008** | **2009** | **2010** | **2011** | **2012** | **2013** | **2014** | **p-value (trend)** | |
|  |  | N (%) | N (%) | N (%) | N (%) | N (%) | N (%) | N (%) | N (%) | N (%) | N (%) | Other trauma | TBI |
| *15 – 17* | Other trauma | 43884  (24.2) | 38644  (24.6) | 36013  (24.8) | 31999  (24.0) | 29656  (23.0) | 27896  (22.4) | 25968  (21.9) | 24469  (21.5) | 22750  (21.4) | 21924  (21.0) |  |  |
|  | TBI | 4902  (24.0) | 4674  (24.4) | 4621  (24.5) | 4333  (23.0) | 4741  (20.6) | 4531  (20.7) | 4410  (20.9) | 4475  (20.0) | 4167  (19.4) | 4018  (19.1) |  |  |
| **Race/Ethnicity** |  |  |  |  |  |  |  |  |  |  |  | <0.001 | <0.001 |
| *Non-Hispanic White* | Other trauma | 67020  (36.9) | 55627  (35.4) | 49091  (33.8) | 43037  (32.3) | 41832  (32.4) | 39403  (31.6) | 37685  (31.8) | 35352  (31.1) | 32126  (30.3) | 31127  (29.7) |  |  |
|  | TBI | 7830  (38.3) | 7114  (37.2) | 6664  (35.3) | 6311  (33.6) | 8043  (35.0) | 7281  (33.2) | 6957  (32.9) | 7204  (32.1) | 6606  (30.7) | 6360  (30.3) |  |  |
| *Non-Hispanic Black* | Other trauma | 19000  (10.5) | 16483  (10.5) | 15339  (10.6) | 14010  (10.5) | 13735  (10.6) | 13324  (10.7) | 12536  (10.6) | 12140  (10.7) | 10994  (10.4) | 10560  (10.1) |  |  |
|  | TBI | 2128  (10.4) | 1897  (9.9) | 1849  (9.8) | 1904  (10.1) | 2318  (10.1) | 2325  (10.6) | 2261  (10.7) | 2377  (10.6) | 2247  (10.4) | 2094  (10.0) |  |  |
| *Hispanic* | Other trauma | 71648  (39.5) | 63724  (40.6) | 61338  (42.2) | 59314  (44.5) | 57731  (44.7) | 55637  (44.7) | 53614  (45.2) | 52028  (45.8) | 49447  (46.6) | 49831  (47.6) |  |  |
|  | TBI | 7634  (37.3) | 7489  (39.1) | 7734  (41.0) | 7989  (42.5) | 9583  (41.7) | 9468  (43.2) | 9197  (43.5) | 9991  (44.6) | 9777  (45.4) | 9813  (46.7) |  |  |
| *Other* | Other trauma | 14971  (8.3) | 13904  (8.9) | 13593  (9.4) | 12475  (9.4) | 12425  (9.6) | 13251  (10.6) | 12362  (10.4) | 11842  (10.4) | 11286  (10.6) | 11175  (10.7) |  |  |
|  | TBI | 1811  (8.9) | 1720  (9.0) | 1724  (9.1) | 1773  (9.4) | 2343  (10.2) | 2292  (10.4) | 2259  (10.7) | 2397  (10.7) | 2393  (11.1) | 2313  (11.0) |  |  |
| *Missing* | Other trauma | 8921  (4.9) | 7253  (4.6) | 6030  (4.2) | 4552  (3.4) | 3432  (2.7) | 2953  (2.4) | 2445  (2.1) | 2231  (2.0) | 2257  (2.1) | 1973  (1.9) |  |  |
|  | TBI | 1050  (5.1) | 916  (4.8) | 887  (4.7) | 829  (4.4) | 682  (3.0) | 578  (2.6) | 448  (2.1) | 453  (2.0) | 492  (2.3) | 417  (2.0) |  |  |
| **Insurance** |  |  |  |  |  |  |  |  |  |  |  | <0.001 | <0.001 |
| *Private* | Other trauma | 76113  (41.9) | 68116  (43.4) | 62606  (43.1) | 58288  (43.7) | 58383  (45.2) | 55399  (44.5) | 53948  (45.5) | 51767  (45.6) | 43861  (41.3) | 40821  (39.0) |  |  |
| **S1 Table. Summary of Patient Characteristics by Year from 2005-2014: Pediatric TBI vs. Other Trauma Index Visits (continued)** | | | | | | | | | | | | | |
|  |  | **2005** | **2006** | **2007** | **2008** | **2009** | **2010** | **2011** | **2012** | **2013** | **2014** | **p-value (trend)** | |
|  |  | N (%) | N (%) | N (%) | N (%) | N (%) | N (%) | N (%) | N (%) | N (%) | N (%) | Other trauma | TBI |
|  | TBI | 8968  (43.9) | 8851  (46.3) | 8704  (46.2) | 8543  (45.4) | 11375  (49.5) | 10398  (47.4) | 10182  (48.2) | 10870  (48.5) | 9752  (45.3) | 8824  (42.0) |  |  |
| *Medicare* | Other trauma | 1361  (0.8) | 591  (0.4) | 640  (0.4) | 654  (0.5) | 450  (0.4) | 729  (0.6) | 645  (0.5) | 778  (0.7) | 721  (0.7) | 823  (0.8) |  |  |
|  | TBI | 152  (0.7) | 56  (0.3) | 66  (0.4) | 79  (0.4) | 66  (0.3) | 137  (0.6) | 144  (0.7) | 169  (0.8) | 206  (1.0) | 173  (0.8) |  |  |
| *Medicaid* | Other trauma | 76483  (42.1) | 63063  (40.2) | 58789  (40.4) | 55506  (41.6) | 53961  (41.8) | 53536  (43.0) | 50939  (42.9) | 49284  (43.4) | 51253  (48.3) | 54246  (51.8) |  |  |
|  | TBI | 8148  (39.8) | 7285  (38.1) | 7246  (38.4) | 7566  (40.2) | 8879  (38.7) | 8951  (40.8) | 8565  (40.6) | 9251  (41.3) | 9610  (44.7) | 10205  (48.6) |  |  |
| *Uninsured* | Other trauma | 15436  (8.5) | 15043  (9.6) | 14630  (10.1) | 12489  (9.4) | 11160  (8.6) | 10411  (8.4) | 9212  (7.8) | 8249  (7.3) | 7483  (7.1) | 6110  (5.8) |  |  |
|  | TBI | 1717  (8.4) | 1674  (8.8) | 1715  (9.1) | 1607  (8.6) | 1662  (7.2) | 1569  (7.2) | 1418  (6.7) | 1322  (5.9) | 1293  (6.0) | 1212  (5.8) |  |  |
| *Other Insured* | Other trauma | 12167  (6.7) | 10178  (6.5) | 8726  (6.0) | 6451  (4.8) | 5201  (4.0) | 4493  (3.6) | 3898  (3.3) | 3515  (3.1) | 2792  (2.6) | 2666  (2.6) |  |  |
|  | TBI | 1468  (7.2) | 1270  (6.6) | 1127  (6.0) | 1011  (5.4) | 987  (4.3) | 889  (4.1) | 813  (3.9) | 810  (3.6) | 654  (3.0) | 583  (2.8) |  |  |
| **Injury Severity Score** |  |  |  |  |  |  |  |  |  |  |  | <0.001 | <0.001 |
| *<9* | Other trauma | 174633  (96.2) | 151045  (96.2) | 139721  (96.1) | 128246  (96.2) | 124289  (96.2) | 120015  (96.4) | 114249  (96.3) | 109358  (96.3) | 102076  (96.2) | 100800  (96.3) |  |  |
|  | TBI | 18893  (92.4) | 17629  (92.1) | 17446  (92.5) | 17507  (93.1) | 21760  (94.7) | 20839  (95.0) | 20170  (95.5) | 21456  (95.7) | 20666  (96.1) | 20206  (96.2) |  |  |
| *9 – 15* | Other trauma | 2178  (1.2) | 1947  (1.2) | 1710  (1.2) | 1590  (1.2) | 1370  (1.1) | 1275  (1.0) | 1132  (1.0) | 1054  (0.9) | 1017  (1.0) | 943  (0.9) |  |  |
|  | TBI | 988  (4.8) | 977  (5.1) | 914  (4.9) | 823  (4.4) | 783  (3.4) | 718  (3.3) | 633  (3.0) | 656  (2.9) | 580  (2.7) | 518  (2.5) |  |  |
| **S1 Table. Summary of Patient Characteristics by Year from 2005-2014: Pediatric TBI vs. Other Trauma Index Visits (continued)** | | | | | | | | | | | | | |
|  |  | **2005** | **2006** | **2007** | **2008** | **2009** | **2010** | **2011** | **2012** | **2013** | **2014** | **p-value (trend)** | |
|  |  | N (%) | N (%) | N (%) | N (%) | N (%) | N (%) | N (%) | N (%) | N (%) | N (%) | Other trauma | TBI |
| *≥16* | Other trauma | 4749  (2.6) | 3999  (2.6) | 3960  (2.7) | 3552  (2.7) | 3496  (2.7) | 3278  (2.6) | 3261  (2.8) | 3181  (2.8) | 3017  (2.8) | 2923  (2.8) |  |  |
|  | TBI | 572  (2.8) | 530  (2.8) | 498  (2.6) | 476  (2.5) | 426  (1.9) | 387  (1.8) | 319  (1.5) | 310  (1.4) | 269  (1.3) | 273  (1.3) |  |  |
| **Injury characteristics (E-code)** |  |  |  |  |  |  |  |  |  |  |  | <0.001 | <0.001 |
| *Penetrating Injury* | Other trauma | 18330  (10.1) | 16340  (10.4) | 14579  (10.0) | 12813  (9.6) | 11705  (9.1) | 11203  (9.0) | 10410  (8.8) | 9829  (8.7) | 9020  (8.5) | 8518  (8.1) |  |  |
|  | TBI | 127  (0.6) | 107  (0.6) | 135  (0.7) | 90  (0.5) | 92  (0.4) | 95  (0.4) | 86  (0.4) | 63  (0.3) | 75  (0.4) | 56  (0.3) |  |  |
| *Falls* | Other trauma | 58110  (32.0) | 51321  (32.7) | 47882  (32.9) | 44303  (33.2) | 43146  (33.4) | 42745  (34.3) | 40788  (34.4) | 39071  (34.4) | 36798  (34.7) | 36481  (34.9) |  |  |
|  | TBI | 8780  (42.9) | 8552  (44.7) | 8398  (44.5) | 8705  (46.3) | 11078  (48.2) | 10960  (50.0) | 10849  (51.4) | 11313  (50.5) | 11006  (51.2) | 10982  (52.3) |  |  |
| *Motor Vehicle Crash* | Other trauma | 8279  (4.6) | 6767  (4.3) | 5865  (4.0) | 4743  (3.6) | 4494  (3.5) | 4291  (3.4) | 3793  (3.2) | 3726  (3.3) | 3320  (3.1) | 3210  (3.1) |  |  |
|  | TBI | 2247  (11.0) | 1914  (10.0) | 1619  (8.6) | 1355  (7.2) | 1351  (5.9) | 1243  (5.7) | 1132  (5.4) | 1177  (5.3) | 1129  (5.3) | 1054  (5.0) |  |  |
| *Other* | Other trauma | 69173  (38.1) | 59888  (38.2) | 54022  (37.2) | 50537  (37.9) | 49181  (38.1) | 48050  (38.6) | 46559  (39.2) | 44817  (39.5) | 41573  (39.2) | 40635  (38.8) |  |  |
|  | TBI | 7154  (35.0) | 6558  (34.3) | 6515  (34.6) | 6337  (33.7) | 8101 | 7950  (36.2) | 7513  (35.6) | 8334  (37.2) | 7723  (35.9) | 7396  (35.2) |  |  |
|  |  |  |  |  |  | (35.3) |  |  |  |  |  |  |  |
| *Missing* | Other trauma | 27668  (15.2) | 22675  (14.4) | 23043  (15.9) | 20992  (15.7) | 20629  (16.0) | 18279  (14.7) | 17092  (14.4) | 16150  (14.2) | 15399  (14.5) | 15822  (15.1) |  |  |
|  | TBI | 2145  (10.5) | 2005  (10.5) | 2191  (11.6) | 2319  (12.3) | 2347  (10.2) | 1696  (7.7) | 1542  (7.3) | 1535  (6.9) | 1582  (7.4) | 1509  (7.2) |  |  |
| **Received care at Level I or II TC** |  |  |  |  |  |  |  |  |  |  |  | <0.001 | <0.001 |
| *No* | Other trauma | 133046  (73.3) | 115796  (73.8) | 109730  (75.5) | 99830  (74.8) | 97121  (75.2) | 94519  (75.9) | 90584  (76.4) | 87498 (77.0) | 81856  (77.1) | 79264  (75.7) |  |  |
| **S1 Table. Summary of Patient Characteristics by Year from 2005-2014: Pediatric TBI vs. Other Trauma Index Visits (continued)^a^** | | | | | | | | | | | | | |
|  |  | **2005** | **2006** | **2007** | **2008** | **2009** | **2010** | **2011** | **2012** | **2013** | **2014** | **p-value (trend)** | |
|  |  | N (%) | N (%) | N (%) | N (%) | N (%) | N (%) | N (%) | N (%) | N (%) | N (%) | Other trauma | TBI |
|  | TBI | 13120  (64.2) | 12577  (65.7) | 12885  (68.3) | 13195  (70.2) | 16767  (73.0) | 16150  (73.6) | 15615 | 16707  (74.5) | 16136  (75.0) | 15367  (73.2) |  |  |
|  |  |  |  |  |  |  |  | (73.9) |  |  |  |  |  |
| *Yes* | Other trauma | 48514  (26.7) | 41195  (26.2) | 35661  (24.5) | 33558  (25.2) | 32034  (24.8) | 30049  (24.1) | 28058  (23.7) | 26095  (23.0) | 24254  (22.9) | 25402  (24.3) |  |  |
|  | TBI | 7333  (35.9) | 6559  (34.3) | 5973  (31.7) | 5611  (29.8) | 6202  (27.0) | 5794  (26.4) | 5507  (26.1) | 5715  (25.5) | 5379  (25.0) | 5630  (26.8) |  |  |
| **Disposition of index visit** |  |  |  |  |  |  |  |  |  |  |  | <0.001 | <0.001 |
| *Discharged (or died in) from ED^b^* | Other trauma | 174395  (96.1) | 150608  (95.9) | 139640  (96.0) | 128263  (96.2) | 124586  (96.5) | 120403  (96.7) | 115060  (97.0) | 110211  (97.0) | 103058  (97.1) | 101718  (97.2) |  |  |
|  | TBI | 18310  (89.5) | 17148  (89.6) | 17100  (90.7) | 17261 | 21524  (93.7) | 20629  (94.0) | 20026  (94.8) | 21347  (95.2) | 20542  (95.5) | 20112  (95.8) |  |  |
|  |  |  |  |  | (91.8) |  |  |  |  |  |  |  |  |
| *Admitted to hospital* | Other trauma | 7165  (4.0) | 6383  (4.1) | 5751  (4.0) | 5125  (3.8) | 4569  (3.5) | 4165  (3.3) | 3582  (3.0) | 3382  (3.0) | 3052  (2.9) | 2948  (2.8) |  |  |
|  | TBI | 2143  (10.5) | 1988  (10.4) | 1758  (9.3) | 1545  (8.2) | 1445  (6.3) | 1315  (6.0) | 1096  (5.2) | 1075  (4.8) | 973  (4.5) | 885  (4.2) |  |  |
| **Died in ED** |  |  |  |  |  |  |  |  |  |  |  | 0.69 | 0.02 |
| *No* | Other trauma | 181534  (100.0) | 156964  (100.0) | 145368  (100.0) | 133366  (100.0) | 129129  (100.0) | 124553  (100.0) | -  - | -  - | -  - | -  - |  |  |
|  | TBI | 20433  (99.9) | 19128  (100.0) | 18852  (100.0) | 18791  (99.9) | 22957  (100.0) | 21939  (100.0) | -  - | -  - | -  - | -  - |  |  |
| *Yes* | Other trauma | 26  (0.0) | 27  (0.0) | 23  (0.0) | 22  (0.0) | 26  (0.0) | 15  (0.0) | -  - | -  - | -  - | -  - |  |  |
|  | TBI | 20  (0.1) | 8  (0.0) | 6  (0.0) | 15  (0.1) | 12  (0.1) | 5  (0.0) | -  - | -  - | -  - | -  - |  |  |
| **Length of stay if admitted (mean/SD)** | Other trauma | 4.05  (17.5) | 3.72  (7.3) | 4.07  (18.3) | 3.75  (9.0) | 3.67  (8.6) | 3.75  (13.5) | 3.8  (12.0) | 4.26  (14.5) | 4.52  (27.5) | 5.46  (69.9) | 0.041 |  |
|  | TBI | 5.39  (15.6) | 5.05  (10.3) | 4.8  (9.7) | 5.17  (11.7) | 4.81  (13.3) | 4.66  (12.3) | 4.23  (9.7) | 4.58  (9.7) | 4.9  (11.7) | 4.25  (7.9) |  | 0.005 |
| **S1 Table. Summary of Patient Characteristics by Year from 2005-2014: Pediatric TBI vs. Other Trauma Index Visits (continued)** | | | | | | | | | | | | | |
|  |  | **2005** | **2006** | **2007** | **2008** | **2009** | **2010** | **2011** | **2012** | **2013** | **2014** | **p-value (trend)** | |
|  |  | N (%) | N (%) | N (%) | N (%) | N (%) | N (%) | N (%) | N (%) | N (%) | N (%) | Other trauma | TBI |
| **Died in Hospital (Inpatient)** |  |  |  |  |  |  |  |  |  |  |  | 0.55 | 0.005 |
| *No* | Other trauma | 7142  (99.7) | 6365  (99.7) | 5731  (99.7) | 5105  (99.6) | 4559  (99.8) | 4156  (99.8) | -  - | -  - | -  - | -  - |  |  |
|  | TBI | 2087  (97.4) | 1942  (97.7) | 1717  (97.7) | 1513  (97.9) | 1421  (98.3) | 1297  (98.6) | -  - | -  - | -  - | -  - |  |  |
| *Yes* | Other trauma | 23  (0.3) | 18  (0.3) | 20  (0.4) | 20  (0.4) | 10  (0.2) | 9  (0.2) | -  - | -  - | -  - | -  - |  |  |
|  | TBI | 56 | 46  (2.3) | 41  (2.3) | 32  (2.1) | 24  (1.7) | 18  (1.4) | -  - | -  - | -  - | -  - |  |  |
|  |  | (2.6) |  |  |  |  |  |  |  |  |  |  |  |

^a^Percentages may not add to 100% due to rounding error. Empty cells are due to lack of death data or lack of any observations for the specified category and year.

^b^Discharged or died in ED includes discharged or transferred to home under care of a Home Intravenous provider from the ED for years 2005 and 2006 only.

Abbreviations: ED – emergency department; SD – standard deviation; TBI – traumatic brain injury; TC – trauma center
