# Supplementary material for: Revisits, readmissions, and outcomes for pediatric traumatic brain injury in California, 2005-2014
Source: PLoS One. 2020 Jan 24;15(1):e0227981. doi: 10.1371/journal.pone.0227981 (PMC6980591; doi:10.1371/journal.pone.0227981)
Supplement: S2 Table — aPercentages may not add to 100% due to rounding error. Empty cells are due to lack of death data or lack of any observations for the specified category and year. be.g. federal, critical access hospital, psychiatric, cancer. ce.g. psychiatric, chemical dependency, physical medicine rehabilitation. Abbreviations: TBI—traumatic brain injury; ED—emergency department; SNF—skilled nursing facility; IRF—intermediate rehabilitation facility; LTCH—long-term care hospital. (DOCX) [file pone.0227981.s005.docx]

**S2 Table. Disposition and Outcomes by Year from 2005-2014: Pediatric TBI vs. Other Trauma Index Visits^a^**

|  |  | **2005** | **2006** | **2007** | **2008** | **2009** | **2010** | **2011** | **2012** | **2013** | **2014** | **Total** |  |  |
| --- | --- | --- | --- | --- | --- | --- | --- | --- | --- | --- | --- | --- | --- | --- |
|  |  | N (%) | N (%) | N (%) | N (%) | N (%) | N (%) | N (%) | N (%) | N (%) | N (%) | N (%) | p-value | p-value  (trend) |
| **Disposition after ED visit** |  |  |  |  |  |  |  |  |  |  |  |  |  |  |
| *Total* | Other trauma | 174395  (96.1) | 150608  (95.9) | 139640  (96.0) | 128263  (96.2) | 124586  (96.5) | 120403  (96.7) | 115060  (97.0) | 110211  (97.0) | 103058  (97.1) | 101718  (97.2) | 1267942  (96.5) | <0.001 | <0.001 |
|  | TBI | 18310  (89.5) | 17148  (89.6) | 17100  (90.7) | 17261  (91.8) | 21524  (93.7) | 20629  (94.0) | 20026  (94.8) | 21347  (95.2) | 20542  (95.5) | 20112  (95.8) | 193999  (93.2) | <0.001 | <0.001 |
| *Died* | Other trauma | 26  (0.0) | 27  (0.0) | 23  (0.0) | 22  (0.0) | 26  (0.0) | 15  (0.0) | -  - | -  - | -  - | -  - | 176  (0.0) | 0.018 | 0.001 |
|  | TBI | 20  (0.1) | 8  (0.1) | 6  (0.0) | 15  (0.1) | 12  (0.1) | 5  (0.0) | -  - | -  - | -  - | -  - | 93  (0.1) | <0.001 | 0.001 |
| *Home* | Other trauma | 170896  (98.0) | 148015  (98.3) | 137192  (98.3) | 127077  (99.1) | 123324  (99.0) | 119309  (99.1) | 113963  (99.1) | 109199  (99.1) | 101907  (98.9) | 100439  (98.7) | 1251321  (98.7) | <0.001 | <0.001 |
|  | TBI | 17837  (97.4) | 16817  (98.1) | 16724  (97.8) | 16953  (98.2) | 21203  (98.5) | 20366  (98.7) | 19769  (98.7) | 21100  (98.8) | 20271  (98.7) | 19781  (98.4) | 190821  (98.4) | <0.001 | <0.001 |
| *Home health service* | Other trauma | 16  (0.0) | 19  (0.0) | 24  (0.0) | 13  (0.0) | 8  (0.0) | 18  (0.0) | 18  (0.0) | 26  (0.0) | 30  (0.0) | 33  (0.0) | 205  (0.0) | <0.001 | <0.001 |
|  | TBI | -  - | 3  (0.0) | 3  (0.0) | 3  (0.0) | 6  (0.0) | 6  (0.0) | 3  (0.0) | 2  (0.0) | 4  (0.0) | 7  (0.0) | 37  (0.0) | 0.40 | 0.13 |
| *SNF, IRF, LTCH, nursing home, supportive care* | Other trauma | 35  (0.0) | 37  (0.0) | 32  (0.0) | 32  (0.0) | 23  (0.0) | 22  (0.0) | 25  (0.0) | 18  (0.0) | 26  (0.0) | 32  (0.0) | 282  (0.0) | 0.45 | 0.50 |
|  | TBI | 9  (0.1) | 9  (0.1) | 11  (0.1) | 3  (0.0) | 11  (0.1) | 5  (0.0) | 5  (0.0) | 7  (0.0) | 4  (0.0) | 8  (0.0) | 72  (0.0) | 0.23 | 0.06 |
| *Other healthcare institution^b^* | Other trauma | 173  (0.1) | 159  (0.1) | 187  (0.1) | 317  (0.3) | 448  (0.4) | 455  (0.4) | 393  (0.3) | 463  (0.4) | 481  (0.5) | 568  (0.6) | 3644  (0.3) | <0.001 | <0.001 |
|  | TBI | 10  (0.1) | 4  (0.0) | 16  (0.1) | 64  (0.4) | 71  (0.3) | 78  (0.4) | 79  (0.4) | 76  (0.4) | 71  (0.4) | 92  (0.5) | 561  (0.3) | <0.001 | <0.001 |
| *Hospice care* | Other trauma | 3  (0.0) | 30  (0.0) | 29  (0.0) | 5  (0.0) | 5  (0.0) | 3  (0.0) | 4  (0.0) | 2  (0.0) | 4  (0.0) | 5  (0.0) | 90  (0.0) | <0.001 | <0.001 |

| **S2 Table. Disposition and Outcomes by Year from 2005-2014: Pediatric TBI vs. Other Trauma Index Visits (continued)** | | | | | | | | | | | | | | | |
| --- | --- | --- | --- | --- | --- | --- | --- | --- | --- | --- | --- | --- | --- | --- | --- |
|  |  | **2005** | **2006** | **2007** | **2008** | **2009** | **2010** | **2011** | | **2012** | **2013** | **2014** | **Total** |  |  |
|  |  | N (%) | N (%) | N (%) | N (%) | N (%) | N (%) | N (%) | | N (%) | N (%) | N (%) | N (%) | p-value | p-value  (trend) |
|  | TBI | 1  (0.0) | 6  (0.0) | 11  (0.1) | 1  (0.0) | -  - | 2  (0.0) | -  - | | -  - | -  - | -  - | 21  (0.0) | <0.001 | <0.001 |
| *Left against medical advice* | Other trauma | 1129  (0.7) | 886  (0.6) | 757  (0.5) | 666  (0.5) | 667  (0.5) | 438  (0.4) | 491  (0.4) | | 363  (0.3) | 431  (0.4) | 478  (0.5) | 6306  (0.5) | <0.001 | <0.001 |
|  | TBI | 237  (1.3) | 172  (1.0) | 190  (1.1) | 196  (1.1) | 215  (1.0) | 145  (0.7) | 139  (0.7) | | 132  (0.6) | 155  (0.8) | 185  (0.9) | 1766  (0.9) | <0.001 | <0.001 |
| *Other* | Other trauma | 2111  (1.2) | 1425  (1.0) | 1390  (1.0) | 128  (0.1) | 80  (0.1) | 140  (0.1) | 151  (0.1) | | 120  (0.1) | 164  (0.2) | 151  (0.2) | 5860  (0.5) | <0.001 | <0.001 |
|  | TBI | 195  (1.1) | 127  (0.7) | 139  (0.8) | 26  (0.2) | 4  (0.0) | 22  (0.1) | 28  (0.1) | | 21  (0.1) | 29  (0.1) | 29  (0.1) | 620  (0.3) | <0.001 | <0.001 |
| *Missing* | Other trauma | 6  (0.0) | 10  (0.0) | 6  (0.0) | 3  (0.0) | 5  (0.0) | 3  (0.0) | 7  (0.0) | | 5  (0.0) | 6  (0.0) | 7  (0.0) | 58  (0.0) | 0.67 | 0.37 |
|  | TBI | 1  (0.0) | 2  (0.0) | -  - | -  - | 2  (0.0) | -  - | -  - | | -  - | 1  (0.0) | 2  (0.0) | 8  (0.0) | 0.39 | 0.86 |
| **Disposition after hospitalization** | |  |  |  |  |  |  |  |  | |  |  |  |  |  |
| *Total* | Other trauma | 7165  (4.0) | 6383  (4.1) | 5751  (4.0) | 5125  (3.8) | 4569  (3.5) | 4165  (3.3) | 3582  (3.0) | 3382  (3.0) | | 3052  (2.9) | 2948  (2.8) | 46122  (3.5) | <0.001 | <0.001 |
|  | TBI | 2143  (10.5) | 1988  (10.4) | 1758  (9.3) | 1545  (8.2) | 1445  (6.3) | 1315  (6.0) | 1096  (5.2) | 1075  (4.8) | | 973  (4.5) | 885  (4.2) | 14223  (6.8) | <0.001 | <0.001 |
| *Home* | Other trauma | 6695  (93.4) | 5945  (93.1) | 5353  (93.1) | 4783  (93.3) | 4291  (93.9) | 3897  (93.6) | 3379  (94.3) | 3156  (93.3) | | 2849  (93.4) | 2736  (92.8) | 43084  (93.4) | 0.30 | 0.72 |
|  | TBI | 1862  (86.9) | 1737  (87.4) | 1529  (87.0) | 1339  (86.7) | 1252  (86.6) | 1169  (88.9) | 952  (86.9) | 953  (88.7) | | 846  (87.0) | 767  (86.7) | 12406  (87.2) | 0.64 | 0.58 |
| *Home health services* | Other trauma | 137  (1.9) | 147  (2.3) | 130  (2.3) | 98  (1.9) | 87  (1.9) | 85  (2.0) | 79  (2.2) | 80  (2.4) | | 64  (2.1) | 72  (2.4) | 979  (2.1) | 0.55 | 0.29 |
|  | TBI | 35  (1.6) | 30  (1.5) | 15  (0.9) | 13 (0.8) | 14  (1.0) | 15  (1.1) | 22  (2.0) | 16  (1.5) | | 19  (2.0) | 15  (1.7) | 194  (1.4) | 0.05 | 0.23 |
| **S2 Table. Disposition and Outcomes by Year from 2005-2014: Pediatric TBI vs. Other Trauma Index Visits (continued)** | | | | | | | | | | | | | | | |
|  |  | **2005** | **2006** | **2007** | **2008** | **2009** | **2010** | **2011** | **2012** | | **2013** | **2014** | **Total** |  |  |
|  |  | N (%) | N (%) | N (%) | N (%) | N (%) | N (%) | N (%) | N (%) | | N (%) | N (%) | N (%) | p-value | p-value  (trend) |
| *Acute care (same hospital)* | Other trauma | 1  (0.0) | 1  (0.0) | 5  (0.1) | -  - | 3  (0.1) | 3  (0.1) | -  - | 3  (0.1) | | -  - | -  - | 16  (0.0) | 0.05 | 0.96 |
|  | TBI | 4  (0.2) | -  - | 1  (0.1) | 1  (0.1) | 2  (0.1) | -  - | 1  (0.1) | -  - | | -  - | -  - | 9  (0.1) | 0.31 | 0.09 |
| *SNF or intermediate care* | Other trauma | 14  (0.2) | 9  (0.1) | 10  (0.2) | 7  (0.1) | 10  (0.2) | 6  (0.1) | 3  (0.1) | 5  (0.2) | | 12  (0.4) | 8  (0.3) | 84  (0.2) | 0.17 | 0.19 |
|  | TBI | 10  (0.5) | 14  (0.7) | 7  (0.4) | 12  (0.8) | 8  (0.6) | 7  (0.5) | 5  (0.5) | 9  (0.8) | | 5  (0.5) | 3  (0.3) | 80  (0.6) | 0.77 | 0.89 |
| *Residential care* | Other trauma | 28  (0.4) | 30  (0.5) | 21  (0.4) | 35  (0.7) | 28  (0.6) | 19  (0.5) | 10  (0.3) | 16  (0.5) | | 12  (0.4) | 24  (0.8) | 223  (0.5) | 0.02 | 0.20 |
|  | TBI | 14  (0.7) | 7  (0.4) | 13  (0.7) | 9  (0.6) | 3  (0.2) | 10  (0.8) | 3  (0.3) | 1  (0.1) | | 1  (0.1) | 4  (0.5) | 65  (0.5) | 0.05 | 0.03 |
| *Other type of hospital care^c^* | Other trauma | 70  (1.0) | 67  (1.1) | 60  (1.0) | 49  (1.0) | 35  (0.8) | 55  (1.3) | 39  (1.1) | 46  (1.4) | | 38  (1.3) | 41  (1.4) | 500  (1.1) | 0.13 | 0.02 |
|  | TBI | 74  (3.5) | 72  (3.6) | 75  (4.3) | 65  (4.2) | 59  (4.1) | 42  (3.2) | 49  (4.5) | 38  (3.5) | | 40  (4.1) | 45  (5.1) | 559  (3.9) | 0.43 | 0.16 |
| *Transferred to acute care (another hospital)* | Other trauma | 135  (1.9) | 123  (1.9) | 117  (2.0) | 98  (1.9) | 79  (1.7) | 67  (1.6) | 49  (1.4) | 54  (1.6) | | 48  (1.6) | 46  (1.6) | 816  (1.8) | 0.32 | 0.01 |
|  | TBI | 71  (3.3) | 68  (3.4) | 61  (3.5) | 63  (4.1) | 72  (5.0) | 47  (3.6) | 38  (3.5) | 41  (3.8) | | 39  (4.0) | 33  (3.7) | 533  (3.8) | 0.42 | 0.31 |
| *Died* | Other trauma | 23  (0.3) | 18  (0.3) | 20  (0.4) | 20  (0.4) | 10  (0.2) | 9  (0.2) | -  - | -  - | | -  - | -  - | 132  (0.3) | 0.76 | 0.20 |
|  | TBI | 56  (2.6) | 46  (2.3) | 41  (2.3) | 32  (2.1) | 24  (1.7) | 18  (1.4) | -  - | -  - | | -  - | -  - | 278  (2.0) | 0.07 | <0.001 |
| *Left against medical advice* | Other trauma | 16  (0.2) | 20  (0.3) | 14  (0.2) | 14  (0.3) | 6  (0.1) | 10  (0.2) | 3  (0.1) | 2  (0.1) | | 10  (0.3) | 5  (0.2) | 100  (0.2) | 0.12 | 0.12 |

| **S2 Table. Disposition and Outcomes by Year from 2005-2014: Pediatric TBI vs. Other Trauma Index Visits (continued)** | | | | | | | | | | | | | | |
| --- | --- | --- | --- | --- | --- | --- | --- | --- | --- | --- | --- | --- | --- | --- |
|  |  | **2005** | **2006** | **2007** | **2008** | **2009** | **2010** | **2011** | **2012** | **2013** | **2014** | **Total** |  |  |
|  |  | N (%) | N (%) | N (%) | N (%) | N (%) | N (%) | N (%) | N (%) | N (%) | N (%) | N (%) | p-value | p-value  (trend) |
|  | TBI | 4  (0.2) | 4  (0.2) | 5  (0.3) | 5  (0.3) | 4  (0.3) | 2  (0.2) | 1  (0.1) | -  - | -  - | 2  (0.2) | 27  (0.2) | 0.59 | 0.16 |
| *Prison/jail* | Other trauma | 25  (0.4) | 13  (0.2) | 7  (0.1) | 14  (0.3) | 10  (0.2) | 9  (0.2) | 8 | 10  (0.3) | 8  (0.3) | 6  (0.2) | 110  (0.2) | 0.49 | 0.69 |
|  |  |  |  |  |  |  |  | (0.2) |  |  |  |  |  |  |
|  | TBI | 3  (0.1) | 3  (0.2) | 2  (0.1) | 3  (0.2) | 3  (0.2) | 3  (0.2) | 1  (0.1) | 2  (0.2) | 3  (0.3) | 2  (0.2) | 25  (0.2) | 0.98 | 0.35 |
| *Blank/missing* | Other trauma | 21  (0.3) | 10  (0.2) | 14  (0.2) | 7  (0.1) | 10  (0.2) | 5  (0.1) | 4  (0.1) | -  - | 5  (0.2) | 2  (0.1) | 78  (0.2) | 0.03 | 0.002 |
|  | TBI | 10  (0.5) | 7  (0.4) | 9  (0.5) | 3  (0.2) | 4  (0.3) | 2  (0.2) | 4  (0.4) | 2  (0.2) | 5  (0.5) | 1  (0.1) | 47  (0.3) | 0.51 | 0.18 |

*^a^*Percentages may not add to 100% due to rounding error. Empty cells are due to lack of death data or lack of any observations for the specified category and year.

*^b^*e.g. federal, critical access hospital, psychiatric, cancer

*^c^*e.g. psychiatric, chemical dependency, physical medicine rehabilitation

Abbreviations: TBI – traumatic brain injury; ED – emergency department; SNF – skilled nursing facility; IRF – intermediate rehabilitation facility; LTCH – long-term care hospital
