# Supplementary material for: Revisits, readmissions, and outcomes for pediatric traumatic brain injury in California, 2005-2014
Source: PLoS One. 2020 Jan 24;15(1):e0227981. doi: 10.1371/journal.pone.0227981 (PMC6980591; doi:10.1371/journal.pone.0227981)
Supplement: S3 Table — Abbreviations: TBI—traumatic brain injury; CI—confidence interval; RH—relative hazard. + p<0.10 * p<0.05 ** p<0.01. (DOCX) [file pone.0227981.s006.docx]

**S3 Table. Negative Binomial Regression Results for Revisit and Readmissions: Pediatric TBI vs. Other Trauma Patients**

|  | **Readmission** | | | | | **Revisit** | | | |
| --- | --- | --- | --- | --- | --- | --- | --- | --- | --- |
|  | **Other Trauma** | | **TBI** | | | **Other Trauma** | | **TBI** | |
|  | RH | 95% CI | | RH | 95% CI | RH | 95% CI | RH | 95% CI |
| *Age (ref. grp.: 15 – 17 years)* | | |  | |  |  |  |  |  |
| 0 – 4 years | 0.26** | 0.21 - 0.33 | | 0.43** | 0.40 - 0.46 | 0.62** | 0.60 - 0.64 | 1.00 | 0.98 - 1.03 |
| 5 – 9 years | 0.25** | 0.22 - 0.27 | | 0.39** | 0.36 - 0.42 | 0.50** | 0.48 - 0.52 | 0.67** | 0.66 - 0.69 |
| 10 – 14 years | 0.63** | 0.59 - 0.68 | | 0.70** | 0.66 - 0.73 | 0.83** | 0.81 - 0.86 | 0.82** | 0.80 - 0.84 |
| *Sex (ref. grp.: Female)* | |  | |  |  |  |  |  |  |
| Male | 0.86** | 0.79 - 0.95 | | 0.70** | 0.67 - 0.73 | 1.08** | 1.05 - 1.11 | 0.84** | 0.83 - 0.85 |
| Missing | 0.42* | 0.20 - 0.86 | | 0.14** | 0.06 - 0.38 | 0.72 | 0.44 - 1.16 | 0.42** | 0.26 - 0.68 |
| *Race/Ethnicity (ref. grp.: Non-Hispanic White)* | | |  | |  |  |  |  |  |
| Non-Hispanic Black | 0.95 | 0.83 - 1.09 | | 1.21** | 1.12 - 1.30 | 0.93** | 0.89 - 0.97 | 1.17** | 1.14 - 1.20 |
| Hispanic | 0.62** | 0.55 - 0.70 | | 0.92** | 0.87 - 0.97 | 0.68** | 0.66 - 0.69 | 0.98** | 0.96 - 0.99 |
| Other | 0.45** | 0.39 - 0.52 | | 0.78** | 0.72 - 0.85 | 0.50** | 0.48 - 0.52 | 0.75** | 0.73 - 0.77 |
| Missing | 0.72** | 0.60 - 0.87 | | 0.87* | 0.76 - 0.98 | 0.71** | 0.67 - 0.76 | 0.84** | 0.80 - 0.88 |
| Median income | 1.00 | 1.00 - 1.00 | | 1.00** | 1.00 - 1.00 | 1.00** | 1.00 - 1.00 | 1.00** | 1.00 - 1.00 |
| (per $1000) |  |  |  |  |  |  |  |  |  |
| *Insurance (ref. grp.: private insurance)* | | |  | |  |  |  |  |  |
| Medicare | 1.40+ | 0.97 - 2.01 | | 1.16 | 0.86 - 1.56 | 1.45** | 1.25 - 1.67 | 1.52** | 1.39 - 1.67 |
| Medicaid | 1.54** | 1.37 - 1.73 | | 1.40** | 1.33 - 1.48 | 1.69** | 1.64 - 1.74 | 1.77** | 1.74 - 1.80 |
| Self-pay/Uninsured | 0.90 | 0.80 - 1.02 | | 0.94 | 0.87 - 1.02 | 1.10** | 1.05 - 1.15 | 1.20** | 1.17 - 1.24 |
| Other | 1.42** | 1.22 - 1.65 | | 1.35** | 1.23 - 1.47 | 1.56** | 1.48 - 1.66 | 1.40** | 1.35 - 1.45 |
| *Injury Severity Scores (ref. grp.: < 9)* | | |  | |  |  |  |  |  |
| 9 – 15 | 1.43* | 1.04 - 1.97 | | 1.42** | 1.27 - 1.58 | 0.80** | 0.70 - 0.92 | 0.78** | 0.75 - 0.82 |
| ≥ 16 | 1.11 | 0.92 - 1.35 | | 3.13** | 2.81 - 3.48 | 1.03 | 0.96 - 1.10 | 0.84** | 0.78 - 0.91 |
| *Injury characteristics (E- codes) (ref. grp.: Any Motor Vehicle Crash)* | | | | | |  |  |  |  |
| Penetrating Injury | 1.26** | 1.09 - 1.46 | | 1.32 | 0.94 - 1.84 | 1.07+ | 0.99 - 1.15 | 1.20* | 1.01 - 1.42 |
| Falls | 0.95 | 0.83 - 1.09 | | 1.10* | 1.02 - 1.19 | 0.95 | 0.89 - 1.02 | 1.15** | 1.11 - 1.20 |
| Other | 1.02 | 0.91 - 1.15 | | 0.97 | 0.90 - 1.04 | 1.07+ | 1.00 - 1.14 | 1.12** | 1.08 - 1.16 |
| Missing | 1.47** | 1.20 - 1.80 | | 1.47** | 1.35 - 1.60 | 1.17** | 1.08 - 1.25 | 1.19** | 1.14 - 1.24 |
| *Received care at level I or II trauma center (ref. grp.: did not receive care at level I or II trauma center)* | | | | | | | | |  |
| Yes | 1.22** | 1.15 - 1.31 | | 1.35** | 1.29 - 1.42 | 0.89** | 0.87 - 0.92 | 0.88** | 0.87 - 0.90 |

Abbreviations: TBI – traumatic brain injury; CI – confidence interval; RH – relative hazard

+ p<0.10 * p<0.05 ** p<0.01
